# ACE2-targeting monoclonal antibody as potent and broad-spectrum coronavirus blocker

**DOI:** 10.1038/s41392-021-00740-y

**Published:** 2021-08-25

**Authors:** Yuning Chen, Ya-Nan Zhang, Renhong Yan, Guifeng Wang, Yuanyuan Zhang, Zhe-Rui Zhang, Yaning Li, Jianxia Ou, Wendi Chu, Zhijuan Liang, Yongmei Wang, Yi-Li Chen, Ganjun Chen, Qi Wang, Qiang Zhou, Bo Zhang, Chunhe Wang

**Affiliations:** 1 Biotherapeutics Discovery Research Center, Shanghai Institute of Materia Medica, Chinese Academy of Sciences, Shanghai, China; 2 University of Chinese Academy of Sciences, Beijing, China; 3 Key Laboratory of Special Pathogens and Biosafety, Wuhan Institute of Virology, Center for Biosafety Mega-ScienceChinese Academy of Sciences, Wuhan, Hubei China; 4 Center for Infectious Disease Research, Westlake Laboratory of Life Sciences and Biomedicine, Key Laboratory of Structural Biology of Zhejiang Province, School of Life Sciences, Westlake University, and Institute of Biology, Westlake Institute for Advanced Study, Hangzhou, Zhejiang China; 5 Beijing Advanced Innovation Center for Structural Biology, Tsinghua-Peking Joint Center for Life Sciences, School of Life Sciences, Tsinghua University, Beijing, China; 6 School of Chinese Materia Medica, Nanjing University of Chinese Medicine, Nanjing, Jiangsu China; 7 Dartsbio Pharmaceuticals, Zhongshan, Guangdong China; 8 Drug Discovery Center for Infectious Disease, Nankai University, Tianjin, China; 9 Fudan University, School of Pharmacy, Shanghai, China

**Keywords:** Infectious diseases, Drug development

## Abstract

The evolution of coronaviruses, such as SARS-CoV-2, makes broad-spectrum coronavirus preventional or therapeutical strategies highly sought after. Here we report a human angiotensin-converting enzyme 2 (ACE2)-targeting monoclonal antibody, 3E8, blocked the S1-subunits and pseudo-typed virus constructs from multiple coronaviruses including SARS-CoV-2, SARS-CoV-2 mutant variants (SARS-CoV-2-D614G, B.1.1.7, B.1.351, B.1.617.1, and P.1), SARS-CoV and HCoV-NL63, without markedly affecting the physiological activities of ACE2 or causing severe toxicity in *ACE2* “knock-in” mice. 3E8 also blocked live SARS-CoV-2 infection in vitro and in a prophylactic mouse model of COVID-19. Cryo-EM and “alanine walk” studies revealed the key binding residues on ACE2 interacting with the CDR3 domain of 3E8 heavy chain. Although full evaluation of safety in non-human primates is necessary before clinical development of 3E8, we provided a potentially potent and “broad-spectrum” management strategy against all coronaviruses that utilize ACE2 as entry receptors and disclosed an anti-coronavirus epitope on human ACE2.

## Introduction

In the last 20 years, coronaviruses have caused three major transmissible disease outbreaks in human, including severe acute respiratory syndrome (SARS),^1^ Middle East respiratory syndrome (MERS)^2^, and coronavirus disease 2019 (COVID-19).^3,4^

One of the challenges to control coronaviruses is that they evolve constantly, even though slower than HIV and influenza.^5^ Analyses of over 28,000 gene sequences of SARS-CoV-2 spike protein (S-protein) in May 2020 revealed a D614G amino acid substitution (SARS-CoV-2-D614G) that was rare before March 2020, but increased greatly in frequency as the pandemic spread worldwide, reaching over 74% of all published sequences by June 2020^6^ and 81% by May 2021 (GISAID). Evolution of coronaviruses renders them ability to evade virus-specific medications.^7,8^ Recently, the emergence of multiple mutant variants of SARS-CoV-2, including B.1.1.7 (UK), B.1.351 (South Africa), P.1 (Brazil)^9^, and B.1.617^10^ (India) manifests such challenge. In fact, a monoclonal antibody against SARS-CoV-2, bamlanivimab, has been revoked Emergency Use Authorization for expected poor performance against variants currently popular in the US (FDA news). In theory, broad-spectrum coronavirus therapeutics can withstand viral mutations and be potentially utilized in future campaigns against different coronavirus outbreaks.

The key to developing broad-spectrum coronavirus therapeutics is to identify broad-spectrum anti-viral targets. Although RNA polymerase is a broad anti-RNA virus target, it suffers from low specificity and efficacy.^11,12^ By employing a multi-dimensional approach, Gordon et al. proposed a set of potential “pan” viral target for coronaviruses, but the druggability of these targets are yet to be evaluated.^13^ ACE2 fusion proteins can act as decoy receptors to trap SARS-CoV-2,^14,15^ but the affinity and developability of these proteins are generally less than antibodies. Recently, Rappazzo et al. generated a set of monoclonal antibodies that bound to a large panel of coronaviruses, but their neutralizing abilities have not been tested yet.^16^

The infection of SARS-CoV-2 is triggered by binding of their envelope spike glycoproteins (S-protein) to angiotensin-converting enzyme 2 (ACE2) molecules expressed on host cells.^17,18^ The S-protein consists of two subunits: (1) S1-subunit (also called S1-protein) at N-terminal, containing the receptor-binding domain (RBD) responsible for ACE2 binding; (2) S2-subunit at C-terminal responsible for membrane fusion.^18^ The RBD of SARS-CoV-2 has been heavily targeted by antibodies as well as small molecule approaches,^19–23^ but the RBD-targeting approaches are prone to drug resistance caused by viral evolution and are not broad-spectrum.

ACE2 is a type-I transmembrane glycoprotein that plays important roles in maintaining blood pressure homeostasis in the renin-angiotensin system.^24,25^ It is a shared receptor for multiple coronaviruses, such as SARS-CoV-2, SARS-CoV, HCoV-NL63,^17,26,27^ bat coronavirus RaTG13,^28^ pangolin coronavirus GX/P2V/2017 and GD/1/2019.^29^ SARS-CoV, a close sibling of SARS-CoV-2 in the coronavirus family, was the culprit that caused SARS outbreak in 2003,^3^ while HCoV-NL63 infects human much more frequently but causes only cold symptoms with moderate clinical impacts.^30^ Binding of coronavirus to ACE2 not only facilitates viral entry into the host cells, but also down-regulates ACE2 expression.^31,32^

Previous results revealed that the RBD-binding site of ACE2 does not overlap with its catalytic site,^33–35^ it is therefore hypothesized that targeting the RBD-binding site on ACE2 with antibodies can block the entry of all ACE2-dependent coronaviruses, while sparing ACE2’s physiological activities. Such antibodies can be theoretically utilized in managing both current and future coronavirus outbreaks and tolerate viral mutations. By targeting ACE2, additionally, the antibody could be evaluated in HCoV-NL63 patients even when COVID-19 patients are no longer available for clinical trials.

To test the hypothesis, we generated a monoclonal antibody, namely 3E8, to target the RBD-binding site on ACE2. The therapeutic potentials and safety profiles of 3E8 were investigated and the key binding sites of 3E8 on human ACE2 molecule were revealed by cryo-EM and mutation studies to aid future drug discovery endeavor.

## Results

### Antibody generation by hybridoma technology

BALB/c mice were immunized with Fc-tagged human ACE2 protein and the sera were screened for binding to ACE2 (supplementary Fig. 1a) and blocking of SARS-CoV-2 S1-subunit and ACE2 interaction (supplementary Fig. 1b). Hybridoma cells were constructed and the supernatants were screened by the same assays mentioned above. Antibody 3E8 was screened out from a pool of neutralizing antibodies as the most efficacious blocker of S1-subunit binding to ACE2. The variable regions of the heavy (V_H_) and light (V_L_) chains were cloned into human IgG4 backbone, transiently expressed in HEK293F cells and purified (supplementary Fig. 1c). Protein qualities were examined by reduced and non-reduced gels (supplementary Fig. 1d).

### 3E8 binds human ACE2 with moderate affinity

**Fig. 1:**
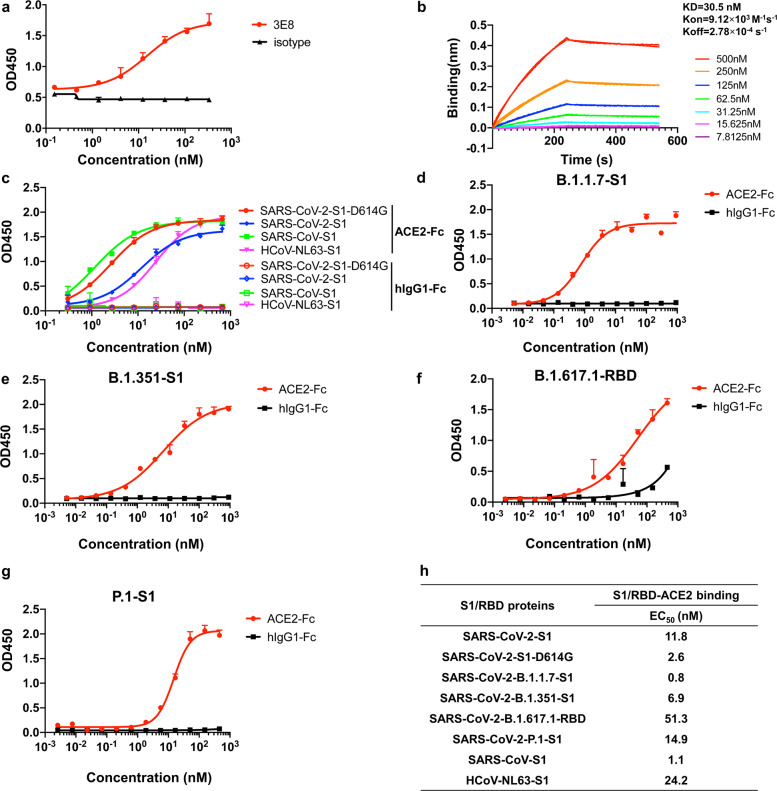
Monoclonal antibody 3E8 and recombinant S1-subunits or RBD from different coronaviruses (and SARS-CoV-2 mutant variants) bound to recombinant human ACE2 protein. **a** Binding of 3E8 to His-tagged recombinant human ACE2 protein as measured by ELISA. **b** Binding of 3E8 to His-tagged human ACE2 as measured by BLI. **c–g** Bindings of recombinant S1-subunits or RBD (in **f** only) from multiple coronaviruses and SARS-CoV-2 mutated variants to Fc-tagged recombinant human ACE2 protein as measured by ELISA. **h** The *EC*_50_ values of recombinant S1-subunit bindings to human ACE2

We measured the binding affinity of 3E8 to His-tagged human ACE2 protein with ELISA and biolayer interferometry (BLI). The *EC*_50_ value was 15.3 nM in ELISA (Fig. 1a) and apparent dissociation constant (KD) was 30.5 nM in BLI using dimeric ACE2 (residues 19-740) as the soluble analyte (Fig. 1b). It also bound to HEK293F cells ectopically overexpressing human ACE2 and to Vero E6 cells endogenously expressing human ACE2, as demonstrated by flow cytometry (supplementary Fig. 1e).

### 3E8 blocks the bindings of S1-subunits or RBD from multiple coronaviruses to ACE2

**Fig. 2:**
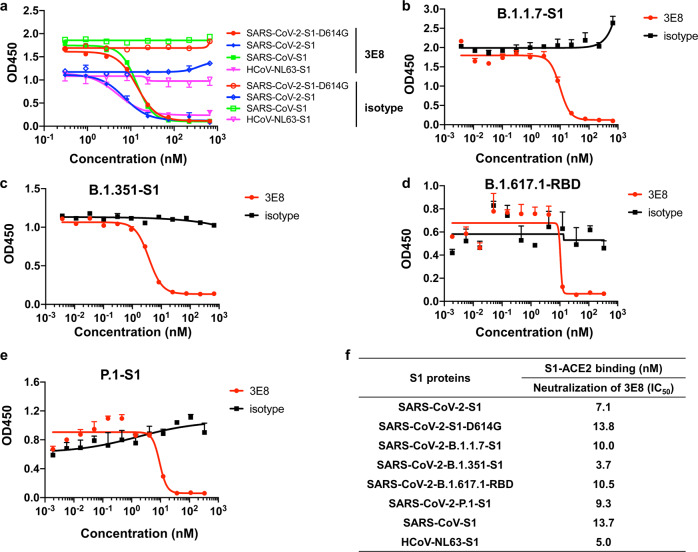
3E8 blocked the bindings of recombinant S1 or RBD from multiple coronaviruses and SARS-CoV-2 variants to Fc-tagged recombinant human ACE2 protein. **a–e** Bindings of His-tagged S1 or RBD (in **d** only) from different coronaviruses including SARS-CoV, HCoV-NL63, SARS-CoV-2, and emerging epidemic SARS-CoV-2 variants to recombinant human ACE2 protein were blocked by 3E8. **f** The *IC*_50_ values of 3E8 in blocking S1 or RBD binding to human ACE2 protein

We investigated the abilities of 3E8 to block the ACE2 binding of S1-subunits or RBD from SARS-CoV-2, SARS-CoV-2-D614G, B.1.1.7, B.1.351, B.1.617.1, P.1, SARS-CoV, and HCoV-NL63. These S1-subunits or RBD can all bind to Fc-tagged human ACE2 molecules (Fig. 1c–g), and the *EC*_50_ values to Fc-tagged recombinant human ACE2 molecule were 11.8, 2.6, 0.8, 6.9, 51.3, 14.9, 1.1, and 24.2 nM, respectively (Fig. 1h). Incubation with 3E8 effectively blocked all S1-subunits or RBD-binding to ACE2 (Fig. 2a–e) and the *IC*_50_ values were 7.1, 13.8, 10.0, 3.7, 10.5, 9.3, 13.7, and 5.0 nM, respectively (Fig. 2f). Thus, 3E8 can broadly block the binding of S1-subunits or RBD from multiple coronaviruses, including the fast-spreading SARS-CoV-2 variants, to human ACE2 molecules.

### 3E8 abolishes the infectivity of multiple pseudo-typed coronaviruses

**Fig. 3:**
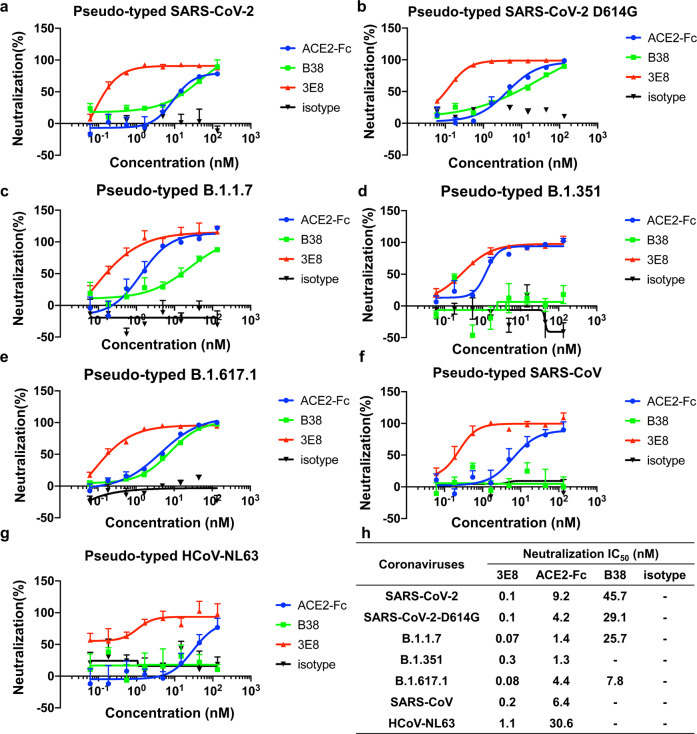
3E8 blocked the infections of ACE2-expressing cells by multiple pseudo-typed coronaviruses. ACE2-Fc and B38 were used as positive controls, and human IgG4 isotype was negative control. **a**–**g** 3E8 blocked infections of ACE2-overexpressing HEK293 cells by different pseudo-typed coronaviruses with Env-defective HIV-1 and full-length S-proteins from SARS-CoV-2, SARS-CoV-2-D614G, B.1.1.7, B.1.351, B.1.617.1, SARS-CoV, and HCoV-NL63. **h** The *IC*_50_ values of 3E8 in blocking pseudo-typed coronaviruses

We next constructed pseudo-typed coronaviruses with full-length S-proteins from SARS-CoV-2, SARS-CoV-2-D614G, B.1.1.7, B.1.351, B.1.617.1, SARS-CoV, and HCoV-NL63 (Fig. 3a–g). All pseudoviruses could infect HEK293F cells that ectopically express human ACE2, while SARS-CoV-2-D614G showed significantly enhanced infectivity when compared to the original SARS-CoV-2 (supplementary Fig. 2). Incubation with 3E8 fully abolished the infectivity of all pseudoviruses, with *IC*_50_ values at 0.1, 0.1, 0.07, 0.3, 0.08, 0.2, and 1.1 nM, respectively (Fig. 3h). In comparison, B38, a SARS-CoV-2 RBD-targeting antibody currently under clinical development,^36^ could only suppress the infectivity of SARS-CoV-2, SARS-CoV-2-D614G, B.1.1.7, and B.1.617.1, but not B.1.351, SARS-CoV, or HCoV-NL63. The suppression by 3E8 was not only broader, but also remarkably more efficacious and potent, as the *IC*_50_ values of 3E8 was hundreds of folds improved when compared to that of B38 (Fig. 3h). ACE2-Fc fusion protein, a virus RBD-targeting molecule consisting the extracellular domain of human ACE2 and the Fc region of human IgG1, showed broad but moderate blocking ability on pseudoviruses. Our investigation indicated that 3E8 was potentially a powerful and broad-spectrum blocker on coronaviruses that are dependent on ACE2.

### 3E8 inhibits live SARS-CoV-2 infection of Vero E6 cells

**Fig. 4:**
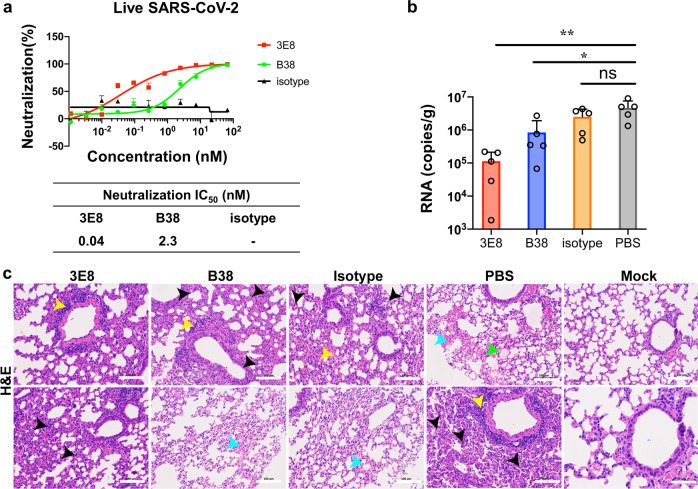
3E8 suppressed the infectivity of live SARS-CoV-2 in Vero E6 cells and a mouse model of COVID-19. **a** 3E8 suppressed the infection of Vero E6 cells by live SARS-CoV-2. **b** Application of 3E8 significantly reduced the viral RNA loads in the lungs of BALB/c mice ectopically expressing human ACE2 and inoculated with live SARS-CoV-2 virus. RBD-targeting monoclonal antibody B38 and isotype were used as positive and negative controls, respectively. **c** H&E staining of lung organ samples from different treatment groups. PBS- and isotype-treated mice developed serious interstitial pneumonia characterized with large area of alveolar septal thickening, large number of inflammatory cell infiltration (black arrow), even formed vascular cuff around blood vessels (yellow arrow), bleeding areas (blue arrow), and material exudates from the alveolar cavity (green arrow). B38-treated mice showed slightly-less histological pneumonia than the two groups. Only inflammatory cell infiltration (black arrow) and formed vascular cuff around blood vessels (yellow arrow) were observed in the lungs of 3E8-treated mice. The scale represents 100 μm. *p < 0.05; **p < 0.01

Live virus study in a BSL-3 laboratory setting showed that incubation with 3E8 inhibited in a concentration-dependent manner the replication of SARS-CoV-2 in Vero E6 cells. The RBD-targeting B38 antibody, also inhibited SARS-CoV-2 replication, but was 60-fold less potent than 3E8, as suggested by the difference between their *IC*_50_ values (2.3 vs. 0.04 nM), even though both completely abolished SARS-CoV-2 replication at high concentrations (Fig. 4a).

### 3E8 blocks SARS-CoV-2 in a prophylaxis mouse model of COVID-19

More importantly, the neutralizing ability of 3E8 was validated in a prophylaxis mouse model of COVID-19. This model was generated by exogenous delivery of hACE2 with Venezuelan equine encephalitis replicon particles, VEEV-VRP-hACE2.^37^ It was a non-lethal infection model that was suitable for measuring viral RNA loads and histological pathology of lungs. After hACE2 was intranasally delivered by VEEV, the antibodies were administered intraperitoneally at 10 mg/kg, and mice were challenged intranasally with 10^5^ PFU of live SARS-CoV-2 12 h later. The viral RNA loads and tissue damages in lungs were examined 3 days post infection. Consistent with our in vitro results, application of 3E8 protected lungs from virus infection, as indicated by ~40-fold reduction in lung viral loads (Fig. 4b) and ameliorated tissue damages (Fig. 4c). In comparison, the viral loads in B38-treated mice were only about 5 times lower than that of control mice. Thus, application of 3E8 achieved significantly greater anti-viral effects than that of B38 in the COVID-19 mouse model we employed.

### 3E8 has no effects on ACE2’s catalytic activities or causes toxicity in “knock-in” mice

**Fig. 5:**
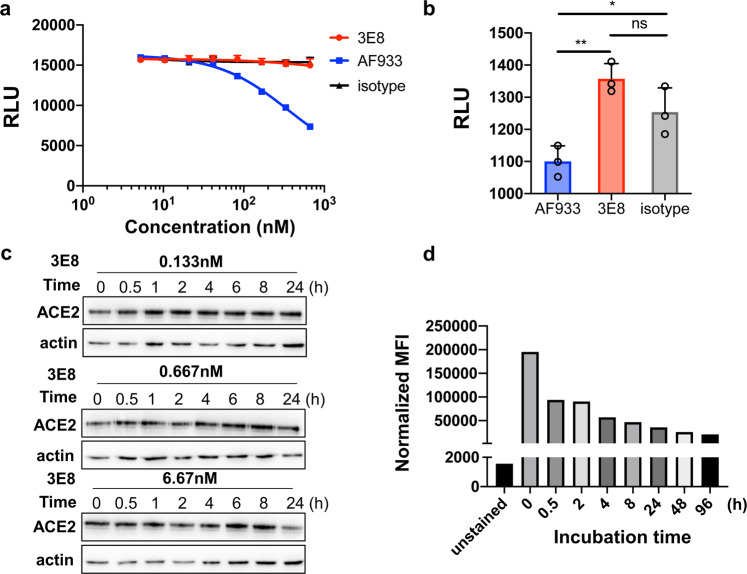
3E8 treatment had no effects on ACE2’s physiological functions. **a** 3E8 treatment had no effects on enzymatic activities of recombinant human ACE2 protein. AF933, an ACE2 polyclonal goat antibody, was used as positive control. hIgG1-Fc and isotype were used as negative controls. **b** 3E8 treatment showed no effects on enzymatic activities of ACE2 molecules endogenously expressed on the surface of Vero E6 cells. **c** Total expression of ACE2 by Vero E6 cells was not affected by 3E8 treatment at 0.133, 0.667, or 6.67 nM for 24 h. **d** The levels of membrane ACE2 expression on HEK293 cells were reduced by 3E8 treatment but were stabilized after 24 h. *p < 0.05; **p < 0.01

Since ACE2 plays important roles in maintaining blood pressure homeostasis in the renin-angiotensin system, we evaluated the safety risks of 3E8 application both in vitro and in vivo. Our studies with both recombinant ACE2 protein and Vero E6 cells suggested that 3E8 had no effects on ACE2’s catalytic activities even at 666.7 nM (Fig. 5a, b). Furthermore, incubation with 3E8 did not trigger a clear trend of ACE2 degradation in Vero E6 cells, as indicated by Western blot (Fig. 5c). Although 3E8 caused time-dependent internalization of ACE2, the levels of membrane-expressed ACE2 were stabilized after 24 h of incubation (Fig. 5d). In limited number of human ACE2 “knock-in” mice, which express human instead of mouse ACE2, injection of 3E8 did not induce noticeable changes in body weights or blood chemistry profiles (supplementary Fig. 3). In addition, there were no obvious differences in shape, size or pathological staining of major organs, including hearts, livers, kidneys, spleens, and lungs of treated mice (supplementary Fig. 3).

### 3E8 binding epitope on ACE2 is determined by cryo-EM and “alanine walk” studies

**Fig. 6:**
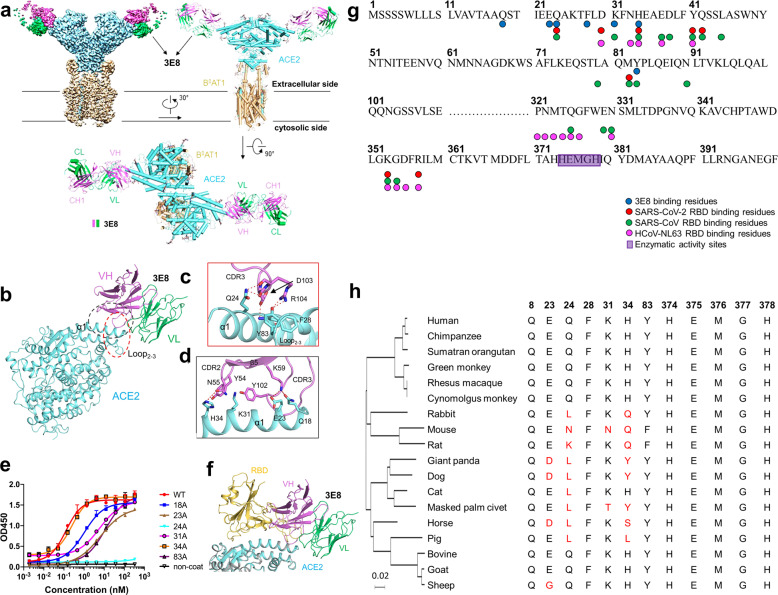
Cryo-EM structure of the 3E8/ACE2-B^0^AT1 complex and “alanine walk” studies to solve the critical interactions between 3E8 and ACE2. Domain-colored cryo-EM map (**a**, upper left panel) and the side view (**a**, upper right panel) or the top view (**a**, lower panel) of the structure. The heavy and light chains of 3E8 are colored green and violet, respectively. The ACE2 and B^0^AT1 are colored cyan and wheat, respectively. **b** Binding interface between 3E8 and ACE2, which contains two clusters that are labeled with red and black dashed ellipses and detailed shown in **c** and **d**, respectively. H-bonds are indicated by red dashed lines. Q (Gln) 24 and H (His)34 on ACE2 were identified as critical amino acid residues interacting with the CDR3 and CDR2 of heavy chain of 3E8, respectively. **e** “Alanine walk” identified Q24 on ACE2 as the most critical amino acid residue for 3E8 binding. **f** Structural alignment of the 3E8/ACE2-B^0^AT1 complex and the RBD/ACE2-B^0^AT1 complex (PDB ID: 6M17) shows clash between 3E8 and RBD of the SARS-CoV-2 S protein. **g** Summary of binding site of multiple coronaviruses on human ACE2. **h** Evolutionary tree of 3E8 binding site on ACE2 with different species

To characterize the epitope recognized by 3E8 on ACE2, we solved the cryo-EM structure of the ACE2-B^0^AT1 complex bound with 3E8 at an overall resolution of 3.2 Å (Fig. 6). Each ACE2 molecule in the complex is bound by a 3E8 molecule that extends from the complex like a wing (Fig. 6a). The heavy chain of 3E8 binds to the peptidase domain of ACE2 mainly through polar interactions between the complementarity-determining region (CDR) 2 and 3 of 3E8 and the N-terminal α1 helix of ACE2 (Fig. 6b). The loop between α2 and α3 of ACE2, referred to as Loop2-3, also contribute limited interactions with 3E8. The resolution at the interface was improved to 3.4 Å by applying focused refinement, supporting detailed analysis on the interactions between ACE2 and 3E8. The interface can be divided into two clusters. At cluster 1, the side chains of Asp103 and Arg104 of 3E8 are hydrogen (H) bonded with the main chain of Phe28 in α1 helix of ACE2 and the side chain of Tyr83 in Loop2-3 of ACE2, respectively (Fig. 6c). Meanwhile, the main chain atoms of Asp103 and Asp104 of 3E8 form H-bonds with the side chain of Gln24 of ACE2. At cluster 2, Tyr54 and Tyr102 of 3E8 interact with Lys31 of ACE2 through cation-π interactions, whereas Asn55 and Lys59 of 3E8 interact with His34 of ACE2 and Glu23 and Gln18 of ACE2, respectively, by forming H-bonds between side chains of these residues (Fig. 6d). Additionally, we performed “alanine walk” studies and identified Gln24 as the most critical amino acid residue that interact with the CDR3 of 3E8 heavy chain (Fig. 6e), consistent with the general concept that the CDR3 of heavy chain contributes the most to antigen recognition and binding. Although His34 was indicated by EM study to interact with the CDR2 of the heavy chain, mutation of it to alanine (Fig. 6e) or other amino acid residues (data not shown) failed to alter the binding of 3E8 to ACE2. Structural alignment of the 3E8/ACE2-B^0^AT1 complex with the previously reported RBD/ACE2-B^0^AT1 complex reveals clash between 3E8 and RBD of SARS-CoV-2 at the binding interface with ACE2 (Fig. 6f), providing an explanation for the results of competition assays. The binding site of 3E8, SARS-CoV-2, SARS-CoV, and HCoV-NL63 on ACE2 were summarized^38–40^ (Fig. 6g). Evolutionary tree of 3E8 binding site on ACE2 with different species was also provided (Fig. 6h), and phylogenetic diversities at position 23, 24, 31, and 34 were identified.

## Discussion

To block the entry of coronavirus, either virus- or ACE2-directed strategies can be taken. Targeting viruses directly is theoretically safer but vulnerable to viral evolution. By targeting ACE2 with an RBD-blocking antibody, we achieved broader and more effective suppression against ACE2-dependent coronaviruses without causing severe side effects. Furthermore, we revealed a broad-spectrum anti-coronavirus epitope on ACE2.

The mechanism by which 3E8 is more potent and efficacious than RBD-targeting antibody B38 is not yet fully understood. Limited by the sample size and evaluation using only a prophylactic regimen in a non-lethal animal model, it is premature to conclude that targeting ACE2 is superior to targeting viral RBD in potency or efficacy. B38 is one of the early anti-SARS-CoV-2 antibodies isolated from COVID-19 patients and due to the urgent nature, it was not well engineered with respect to affinity and developability. More head-to-head studies with more ACE2- and RBD-targeting molecules are necessary before drawing any conclusion.

ACE2-Fc (or called ACE2-Ig) fusion protein molecules may act as a “decoy” to interfere coronaviruses from binding to endogenous ACE2 molecules (Figs. 2 and 3). Although ACE2-Fc molecules are broad-spectrum in theory, their binding affinity (to RBDs), specificity, and developability are usually lower than antibodies. ACE2-Fc was included in our studies as a control and moderate efficacy was observed in vitro. Thus, ACE2-neutralizing antibody appears to be a more favorable approach than ACE2-Fc fusion protein.

“Cocktails” or combination therapies have been currently explored in treating COVID-19.^19^ A combination of 3E8 with antibodies recognizing different epitopes (e.g., RBD, NTD, and/or glycan) on the viral surface seems a viable option and could be explored in clinic for better efficacy.

It is not surprising that no severe side effects or toxicity of 3E8 were observed in vitro or in human *ACE2* “knock-in” mice. In vitro, 3E8 did not affect the catalytic activities of ACE2 or trigger significant ACE2 down-regulation. Even though ACE2 internalization was overserved, the levels of membrane ACE2 expression were stabilized after 24 h. It is possible that the ACE2 molecules remaining on the membrane are sufficient to maintain the physiological functions of ACE2-expressing cells. Previous studies showed that ACE2 “knockout” mice were viable and healthy in general, even though the contractile dysfunction was found,^41^ indicating that ACE2 is not crucial to the survival of animals. Due to limited animal availability, the conclusion from human *ACE2* “knock-in” mice should not be overinterpreted. We plan to repeat this study when more animals are commercially available. Moreover, key signs of cardiovascular health, such as pulse pressure and heartbeat rate, cannot be conveniently measured in mice. Thus, the side effects and toxicities of 3E8 should be carefully evaluated in non-human primates before moving to the clinic.

A few broad-spectrum anti-coronavirus antibody or virus decoy receptor strategies have been disclosed.^14–16^ Among them, Rappazzo et al.^16^ reported an RBD-targeting antibody with exceptional breadth against distantly related SARS viruses, even though some highly transmissible variants were not explicitly tested since they were not well described in January 2021 when Rappazzo’s manuscript was accepted for publication. The antibody performed well in a phase 1 trial and is currently in a phase 2/3 trial, exemplifying the power of broad-spectrum antibody therapies. Nevertheless, current coronavirus-targeting antibodies focus mainly on highly conserved regions of RBD, such as S309,^20^ 47D11,^21^ D405, G502, G504, and Y50.^16^ The epitope of 3E8 binding on ACE2 is only partially overlapping with that of RBD domain, but blocked virus infections with remarkable efficiency, demonstrating the extraordinary power of ACE2 targeting strategy. Previously, neutralizing antibodies (4A8)^42^ and 89C8-ACE2^43^ were isolated from convalescent COVID-19 patients with binding on the N-terminal domain (NTD) of the SARS-CoV-2 S-protein, but not the RBD. Our results highlighted again the importance of epitope outside or on the verge of RBD/ACE2 interface, and would facilitate future endeavor searching for broad-spectrum anti-coronavirus approaches.

Overall, we presented evidence that 3E8 was a promising therapeutic candidate for coronavirus pandemic and believe that it represents a significant conceptual advance in fighting COVID-19, which keeps evolving, and may open the door for more ACE2-targeting drug discovery and development.

## Materials and methods

### Spike mutations on SARS-CoV-2 variants

B.1.1.7: H69 deletion, V70 deletion, Y144deletion, N501Y, A570D, D614G, P681H, T716I, S982A, D1118H;

B.1.351: D80A, D215G, A242-244 deletion, K417N, E484K, N501Y, D614G, A701V;

B.1.617.1: T95I, G142D, E154K, L452R, E484Q, D614G, P681R, Q1071H;

P.1: L18F, T20N, P26S, D138Y, R190S, K417T, E484K, N501Y, D614G, H655Y, T1027I, V1176F.

### Biolayer interferometry

Binding affinities were measured by BLI using Fortebio Octet Red 96. For affinity measurement, 10 μg/ml of 3E8 was captured by protein A biosensor and incubated with different concentrations of hACE2-his protein. The baseline was established by PBS with 0.05% tween-20 for 60 s. The association was set at 240 s and the dissociation periods was set at 300 s. The mean Kon, Koff, and apparent KD values of binding affinities were calculated from all binding curves based on their global fit to 1:1.

### Neutralization ELISA

S1-proteins (2 μg/ml) were coated onto plates at 4 °C overnight. Serial dilutions of 3E8 or isotype were pre-incubated with 5 μg/ml of ACE2-Fc for 30 min at room temperature. Then, the mixture was added into the coated plate wells and incubated for 1 h. The bound ACE2-Fc was detected by HRP-conjugated goat anti-human IgG and developing substrate.

### Pseudo-typed virus neutralization assay

Pseudo-typed SARS-CoV-2-D614G, SARS-CoV-2, SARS-CoV, HCoV-NL63, B.1.1.7, B.1.351 and B.1.617.1 were constructed by co-transfection of two plasmids, one expressing Env-defective HIV-1 with luciferase reporter (pNL4-3.luc.RE)^44^ and the other expressing the full-length S-protein of SARS-CoV-2-D614G, SARS-CoV-2, SARS-CoV, HCoV-NL63, B.1.1.7, B.1.351, or B.1.617.1 into HEK293T cells. The supernatant containing virus particles was harvested 48 h post-transfection followed by 0.45 μm filtration. HEK293F/ACE2/EGFP cells were pre-seeded with 1.2 × 10^4^ cells per well in a 96-well plate. The confluent cells were incubated with 50 μl of serially diluted antibodies or ACE2-Fc for 1 h at 37°C followed by addition of various pseudoviruses of the same volume. In all, 100 μl of DMEM with 10% FBS was added as negative control. In total, 100 μl of pseudovirus and DMEM mixed at ratio 1:1 was used as positive control. After 24 h, medium was changed and the cells were incubated for another 48 h. The relative light units (RLUs) of luminescence were measured by Firefly Luciferase Reporter Assay Kit (Meilunbio). Neutralization (%) = [1−(RLU_samples_−RLU_negtive control_)/(RLU_positive control_−RLU_negtive control_)] ×100%. The *IC*_50_ values were calculated by non-linear.

### Live SARS-CoV-2 suppression in vitro

Vero E6 (ATCC® CRL-1586™) cells were trypsinized and pre-seeded into 24-well plates in duplicate with 1 × 10^5^ cells/well in DMEM containing 10% FBS (100 U/mL of penicillin and 100 μg/ml of streptomycin) at 37 °C with 5% CO_2_ 1 day before. After confluent, antibodies of 3-fold serially diluted were added, and Vero E6 cells were infected with live SARS-CoV-2 (IVCAS 6.7512) virus at a multiplicity of infection (MOI) of 0.01. After 24 h incubation, the culture supernatants were collected and viral RNA was quantified via qRT-PCR using Luna® Universal Probe One-Step RT-PCR Kit (E3006) on CFX96 Touch^TM^ Real-Time PCR Detection System (Bio Rad). Primers used were as follows: RBD-qF1: 5′-caatggtttaacaggcacagg-3′, RBD-qR1: 5′-ctcaagtgtctgtggatcacg-3′, Probe: acagcatcagtagtgtcagcaatgtctc. *IC*_50_ was fitted and calculated by GraphPad Prism 8. Data represents as mean ± SD of two replicates from one representative experiment, and the experiment was repeated for thre times.

### COVID-19 mouse model and disease suppression

BALB/c mice were purchased from Wuhan Institute of Biological Products Co. Ltd. and cared in accordance with the recommendations of National Institutes of Health Guidelines for the Care and Use of Experimental Animals. All the animal studies were conducted in biosafety level 3 (BSL-3) facility at Wuhan Institute of Virology under a protocol approved by the Laboratory Animal Ethics Committee of Wuhan Institute of Virology, Chinese Academy of Sciences (Permit number: WIVA26201701).

A mouse model recently established by VEEV-VRP delivery of ACE2 for SARS-CoV-2 infection^37^ was used to evaluate the efficacy of 3E8 in vivo. Four groups of 6–8-week-old female BALB/c mice (*n* = 5 per group) were first intranasally infected with 10^6^ FFU VRP-ACE2 per mouse in a total volume of 80 μl after anesthetization with Avertin (250 mg/kg). The mice from different groups were treated with 3E8, B38 or isotype at a dose of 10 mg/kg 12 h later via intraperitoneal injection. After another 12 h, all mice were intranasally infected with 10^5^ PFU SARS-CoV-2 in a total volume of 50 μl. Three days post infection of SARS-CoV-2, the lungs of mice were collected for viral RNA quantification and histological analysis. For RNA quantification, some lungs were homogenized in DMEM medium, and viral RNA was extracted using QIAamp viral RNA mini kit (52906, Qiagen) following the manufacturer’s protocol. qRT-PCR assay was performed using Luna® Universal Probe One-Step RT-PCR Kit (E3006). Primers and probe used were: RBD-qF1: 5′-caatggtttaacaggcacagg-3′, RBD-qR1: 5′- ctcaagtgtctgtggatcacg -3′, Probe: acagcatcagtagtgtcagcaatgtctc. For histological analysis, lung samples from mice were fixed with 4% paraformaldehyde, embedded in paraffin, sagittally sectioned at 4 μm thickness on a microtome, and mounted on APS-coated slides for H&E stain.

### ACE2 enzymatic activity assay

The catalytic activities of recombinant and endogenous ACE2 was detected according to a published protocol using fluorescent substrate, Mca-APK-Dnp (AnaSpec).^45^ To determine the impact on enzyme activity, serial diluted antibodies were pre-incubated with 2.0 μg/ml of recombinant human ACE2 at room temperature for 1 h on a shaker. After incubation, the neutralization solution was 1:5 diluted with activity buffer^45^ and then mixed with 50 μl/well of 200 mM substrate. The mixture was incubated at 37 °C for 20 min before the RFU of fluorescent signals were read on an Envision microplate reader (Perkin Elmer, Waltham, MA) with excitation wavelength set at 320 nm and emission wavelength set at 400 nm. To measure endogenous ACE2 enzymatic activity, Vero E6 cells were seeded in a 96-well plate at 1 × 10^5^ cells/well and cultured overnight. The cells were then incubated with 10 μg/ml of 3E8, AF933 (R&D Systems, Minneapolis, MN) and isotype for 60 min at 37 °C before mixed with 50 μl/well of activity buffer and 50 μl/well of substrate. The cells were incubated for 20 min at 37 °C before transferred to black 96-well plate for fluorescence reading.

## Supplementary information

210814 supplementary-clean version plain text final

## Data Availability

All data supporting the findings of this study are available from the corresponding author on reasonable request.
