# Supplementary material for: ACE2-targeting monoclonal antibody as potent and broad-spectrum coronavirus blocker
Source: Signal Transduct Target Ther. 2021 Aug 25;6:315. doi: 10.1038/s41392-021-00740-y (PMC8385704; doi:10.1038/s41392-021-00740-y)
Supplement: Supplementary file 1 — 210814 supplementary-clean version plain text final [file 41392_2021_740_MOESM1_ESM.docx]

Supplementary Materials for

ACE2-Targeting Monoclonal Antibody as Potent and Broad-Spectrum Coronavirus Blocker

Yuning Chen**^1,7,^***, Yanan Zhang**^2,7,^***, Renhong Yan**^3,^***, Guifeng Wang**^1,^***, Yuanyuan Zhang**^3^**, Zherui Zhang**^2,7^**, Yaning Li**^5^**, Jianxia Ou**^8^**, Wendi Chu**^1,7^**, Zhijuan Liang**^1,7^**, Yongmei Wang**^1,7^**, Yili Chen**^4^**, Ganjun Chen**^4^**, Qi Wang**^1^**, Qiang Zhou**^3^**†, Bo Zhang**^2,6^**†, Chunhe Wang**^1,4,7,8,9^**†

Chunhe Wang ([wangc@simm.ac.cn](mailto:wangc@simm.ac.cn)), Bo Zhang ([zhangbo@wh.iov.cn](mailto:zhangbo@wh.iov.cn)) or Qiang Zhou (zhouqiang@westlake.edu.cn).

**This PDF file includes:**

Additional Materials and Methods

Figures. S1 to S6

Table S1

References and notes

**Materials and Methods**

Protein expression and purification

The His-tagged S1 proteins of SARS-CoV, HCoV-NL63, SARS-CoV-2, SARS-CoV-2 D614G, B.1.17, B.1.351, P.1 and the His-tagged RBD protein of B.1.617.1 were purchased from Sino Biological (Beijing, China). ACE2-his, ACE2-Fc and 3E8 were prepared as below: The DNA fragments encoding extracellular ACE2 domain (residues 19-740) from plasmid encoding full-length ACE2 (Sino Biological) were subcloned into the mammalian expression vectors pTT5 and pINFUSE (Vivogen, San Diego, CA) with C-terminal 6×His or hIgG1-Fc tags. The codon-optimized variable regions of the heavy and light chain of 3E8 were cloned into expression vectors containing human IgG4 constant regions. Purified plasmids were transfected into HEK293F cells (Shanghai Cell Line Bank, China) by polyethylenimine (Polysciences, Warrington, PA). Cells were then cultured in suspension in CD medium. After 5 days of culture, the supernatant was collected and purified by Ni-NTA or protein A chromatography. Size exclusion chromatography column (SEC) was used to examine the purity of proteins.

Binding ELISA

96-well Immuno-plates (Greiner) were coated with 2.0 µg/ml of purified recombinant 6×His-tagged human ACE2 protein at 4℃ overnight. After blocking at room temperature for 1 h with 1% casein (Thermo Fisher), plates were washed with PBS containing 0.05% Tween-20, and serial dilutions of 3E8 were added for 1 h incubation. After washing, goat anti-human IgG conjugated with HRP (AB Clonal Technology, 1:2000 dilution) was added and incubated for 1 h, then TMB substrate (Thermo Fisher Scientific) and 2 M of H_2_SO_4_ were added, and OD_450_ was detected with SpectraMax M5e (Molecular Devices) microplate reader. To measure the binding affinity of S1 proteins to ACE2, 2 μg/ml of various S1 proteins were coated onto plates followed by addition of gradient diluted ACE2-Fc, and goat anti-human IgG conjugated with HRP was used for detection.

Flow Cytometry

Vero E6 and HEK293/ACE2/EGFP cells were harvested and aliquoted into FACS tubes at 5×10^5^ cells/tube. The cells were washed with cold staining buffer (PBS+0.1% BSA+0.04% Na3N) and then resuspended in 100 μl of 3E8 at different concentrations. The cells were kept at 4℃ in the dark for 1 h on a shaker before washed twice. The cells were resuspended in 100 μL of staining buffer containing PE-goat-anti-human IgG (Biolegend) at 4 μg/ml for 30 min. The cells were washed twice and resuspended in 200 μl of staining buffer for flow cytometry analysis.

Western Blot

Vero E6 cells were incubated at 37℃ with different concentrations of 3E8 in DMEM with 10% FBS and lysed with commercial cell lysis buffer (Beyotime, Shanghai, China) at different time points. Western blot analysis for ACE2 protein in whole cell lysate was carried out using rabbit anti-ACE2 pAb (1:500, Sino biological) and goat anti-rabbit conjugated with HRP (1:2000, Abclonal, Wuhan, China) using a standard Western blot protocol.

Cryo-EM sample preparation

The ACE2-B^0^AT1 complex was mixed with 3E8 at a molar ratio of 1:1.5 at 4 °C for 1hr before applied to the grids. Aliquots (3.3 μl) of the protein complex were placed on glow-discharged holey carbon grids (Quantifoil Au R1.2/1.3). The grids were blotted for 2.5 s or 3.0 s and flash-frozen in liquid ethane cooled by liquid nitrogen with Vitrobot (Mark IV, Thermo Scientific). The cryo-EM samples were transferred to a Titan Krios operating at 300 kV equipped with Cs corrector, Gatan K3 Summit detector and GIF Quantum energy filter. Movie stacks were automatically collected using AutoEMation ^1^, with a slit width of 20 eV on the energy filter and a defocus range from -1.2 µm to -2.2 µm in super-resolution mode at a nominal magnification of 81,000×. Each stack was exposed for 2.56 s with an exposure time of 0.08 s per frame, resulting in a total of 32 frames per stack. The total dose rate was approximately 50 e^-^/Å^2^ for each stack. The stacks were motion corrected with MotionCor2 ^2^ and binned 2-fold, resulting in a pixel size of 1.087 Å/pixel. Meanwhile, dose weighting was performed ^3^. The defocus values were estimated with Gctf ^4^.

Cryo-EM data processing

Particles were automatically picked using Relion 3.0.6 ^5-8^ from manually selected micrographs. After 2D classification with Relion, good particles were selected and subject to two cycle of heterogeneous refinement without symmetry using cryoSPARC ^9^. The good particles were selected and subjected to Non-uniform Refinement (beta) with C1 symmetry, resulting in the 3D reconstruction for the whole structures, which was further subject to 3D classification, 3D auto-refinement and post-processing with Relion with C2 symmetry. To further improve the map quality for interface between 3E8 and ACE2-B^0^AT1 complex, the particles were C2-symmetry expanded and re-centered at the location of the interface between 3E8-ACE2 sub-complex. The re-extracted dataset was subject to focused refinement with Relion, resulting in the 3D reconstruction of better quality on the binding interface.

The resolution was estimated with the gold-standard Fourier shell correlation 0.143 criterion ^10^ with high-resolution noise substitution ^11^. Refer to Supplementary Figures S4-S6 and Supplementary Table 1 for details of data collection and processing.

Cryo-EM model building and structure refinement

For model building of 3E8 bound with ACE2-B^0^AT1 complex, the atomic model of the published structure S-ECD (PDB ID: 7C2L) and ACE2-B^0^AT1 complex (PDB ID: 6M18) were used as templates, which were molecular dynamics flexible fitted (MDFF) ^12^ into the whole cryo-EM map of the complex and the focused-refined cryo-EM map of the 3E8-ACE2 sub-complex, respectively. And the fitted atomic models were further manually adjusted with Coot ^13^. Each residue was manually checked with the chemical properties taken into consideration during model building. Several segments, whose corresponding densities were invisible, were not modeled. Structural refinement was performed in Phenix ^14^ with secondary structure and geometry restraints to prevent overfitting. To monitor the potential overfitting, the model was refined against one of the two independent half maps from the gold-standard 3D refinement approach. Then, the refined model was tested against the other map. Statistics associated with data collection, 3D reconstruction and model building were summarized in supplementary Table 1.

“Alanine walk” studies

To verify the key residues of ACE2 on binding with 3E8, Q18, E23, Q24, F28, K31, H34 and Y83 on ACE2 were changed into alanine using the overlapped PCR method with PrimeSTAR HS DNA Polymerase (Takara) and the following primers. Plasmids with mutation were expressed in mammalian expression system. After purification by Ni-NTA affinity chromatography, proteins were checked by SDS-PAGE and ELISA.

| **Primers** | | **Sequence** |  |
| --- | --- | --- | --- |
| 18A | F | GGGTGCACTCCGCGTCCACCATTG |  |
|  | R | CAATGGTGGACGCGGAGTGCACCC |  |
| 23A | F | CCATTGAGGCACAGGCCAAGAC |  |
|  | R | GTCTTGGCCTGTGCCTCAATGG |  |
| 24A | F | CATTGAGGAAGCGGCCAAGACATTTTTG | |
|  | R | CAAAAATGTCTTGGCCGCTTCCTCAATG | |
| 28A | F | GGCCAAGACAGCGTTGGACAAGTTTAAC | |
|  | R | GTTAAACTTGTCCAACGCTGTCTTGGCC | |
| 31A | F | GACATTTTTGGACGCGTTTAACCACGAAGC | |
|  | R | GCTTCGTGGTTAAACGCGTCCAAAAATGTC | |
| 34A | F | CAAGTTTAACGCCGAAGCCGAAGAC | |
|  | R | GTCTTCGGCTTCGGCGTTAAACTTG | |
| 83A | F | CTTGCCCAAATGGCGCCACTACAAGAAATTC | |
|  | R | GAATTTCTTGTAGTGGCGCCATTTGGGCAAG | |
| 34G | F | CAAGTTTAACGGCGAAGCCGAAGAC | |
|  | R | GTCTTCGGCTTCGCCGTTAAACTTG | |
| 34W | F | CAAGTTTAACTGGGAAGCCGAAGAC | |
|  | R | GTCTTCGGCTTCCCAGTTAAACTTG | |
| 34K | F | CAAGTTTAACAAGGAAGCCGAAGAC | |
|  | R | GTCTTCGGCTTCCTTGTTAAACTTG | |
| 34D | F | CAAGTTTAACGACGAAGCCGAAGAC | |
|  | R | GTCTTCGGCTTCGTCGTTAAACTTG |  |

Toxicity studies

Five-week-old male human ACE2 “knock-in” mice on C57BL/6 background were purchased from Shanghai Model Organisms Center (Shanghai). Animal handling and procedures were approved and performed according to the requirements of the Institutional Animal Care and Use Committee (IACUC) of Shanghai Institute of Materia Medica. Five male mice were randomized into two groups: 3E8 (3 mice, 9#, 66# and 86#) and isotype (2 mice, 68# and 39#). The mice received an intravenous (i.v.) injection of 100 μl (30 mg/kg) of 3E8 or isotype. The mice were weighed and assessed for behavioral changes at 0, 24, 72 and 144 h time points after injection. Toxicities was evaluated by body weight measuring, serum biochemistry and pathology studies. After 7 days of treatment, all mice were sacrificed by cervical dislocation. Blood, hearts, livers, spleens, lungs and kidneys were collected for biochemistry and pathology studies. Organs were fixed with 10% buffered formalin and subjected to paraffin embedding before sectioned, deparaffined, rehydrated and stained with Hematoxylin and Eosin (H&E) staining.

Statistical analysis

Data were shown as mean ± SEM or SD. Statistical difference were calculated by Student’s t-test, with **P* < 0.05 considered significant and *P* < 0.01 highly significant.


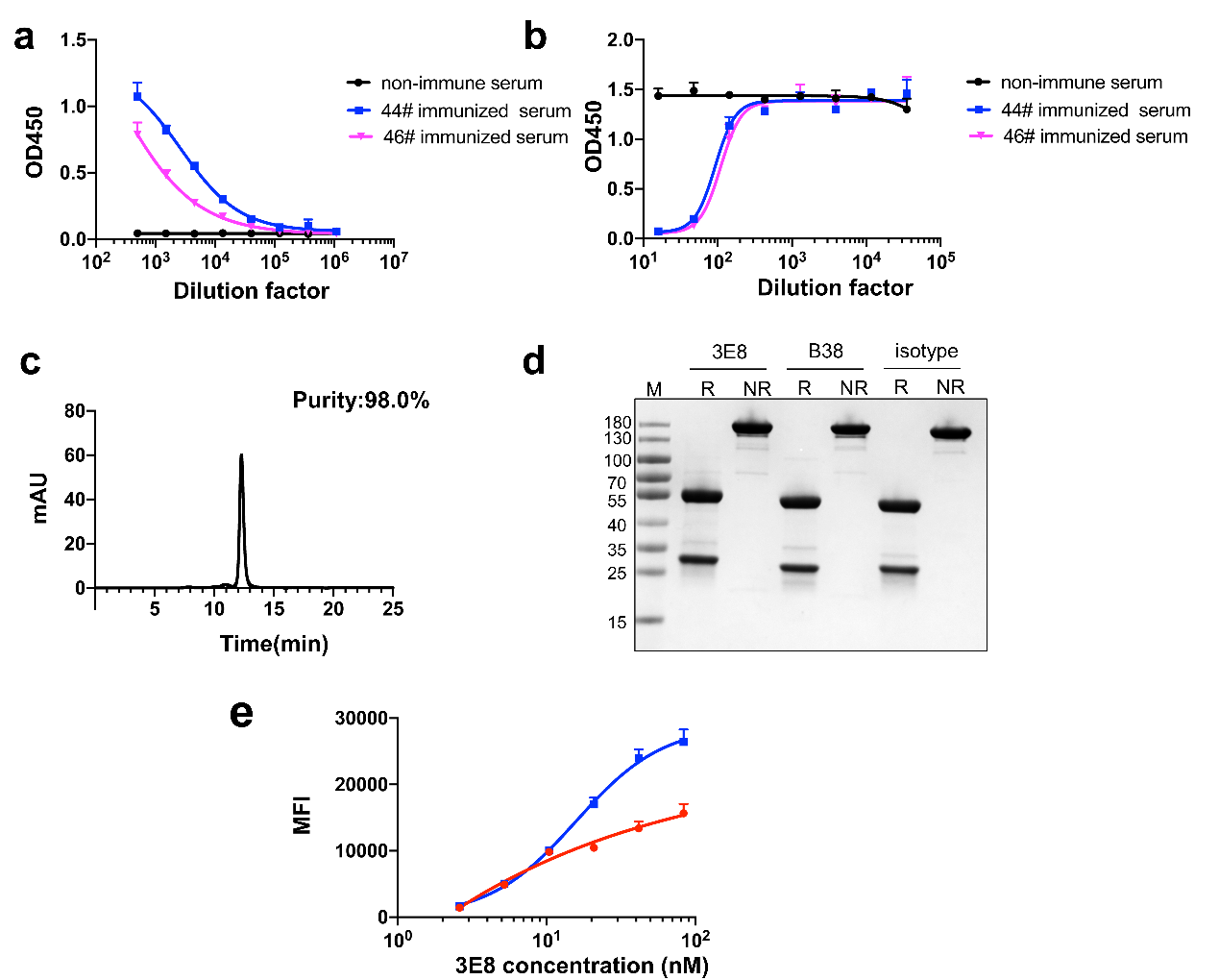


**Figure. S1.**

Bindings of immunized mice sera and purified 3E8 to recombinant human ACE2 protein. **a** Sera from ACE2-immunized mice bound to His-tagged recombinant human ACE2 protein. **b** Sera from ACE2-immunized mice blocked binding of SARS-CoV-2 S1-subunit to His-tagged recombinant ACE2 protein. 44# and 46# in A and B are individual immunized mice. **c** SEC profile of 3E8 on MAbPac SEC-1 column. The flow rate was 0.2 ml/min, the mobile phase was PBS buffer, and the monomer retention time was 12.28 min. **d** SDS-PAGE gels of the 3E8. R: reduced; NR: non-reduced. M: marker **e** Bindings of 3E8 to Vero E6 and HEK293 cells expressing human ACE2 measured as analyzed by flow cytometry.


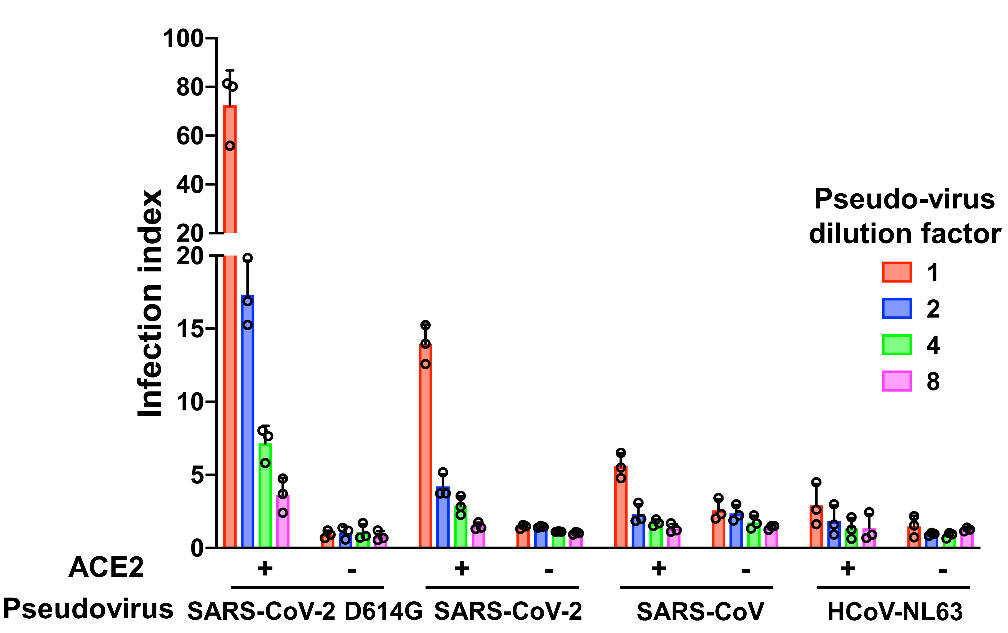


**Figure. S2.**

Infection of HEK293F/ACE2/EGFP cells by different pseudo-typed coronaviruses. Pseudo-typed SARS-CoV-2-D614G, SARS-CoV-2 (D614), SARS-CoV and HCoV-NL63 were constructed and infected HEK293 cells overexpressing human ACE2.


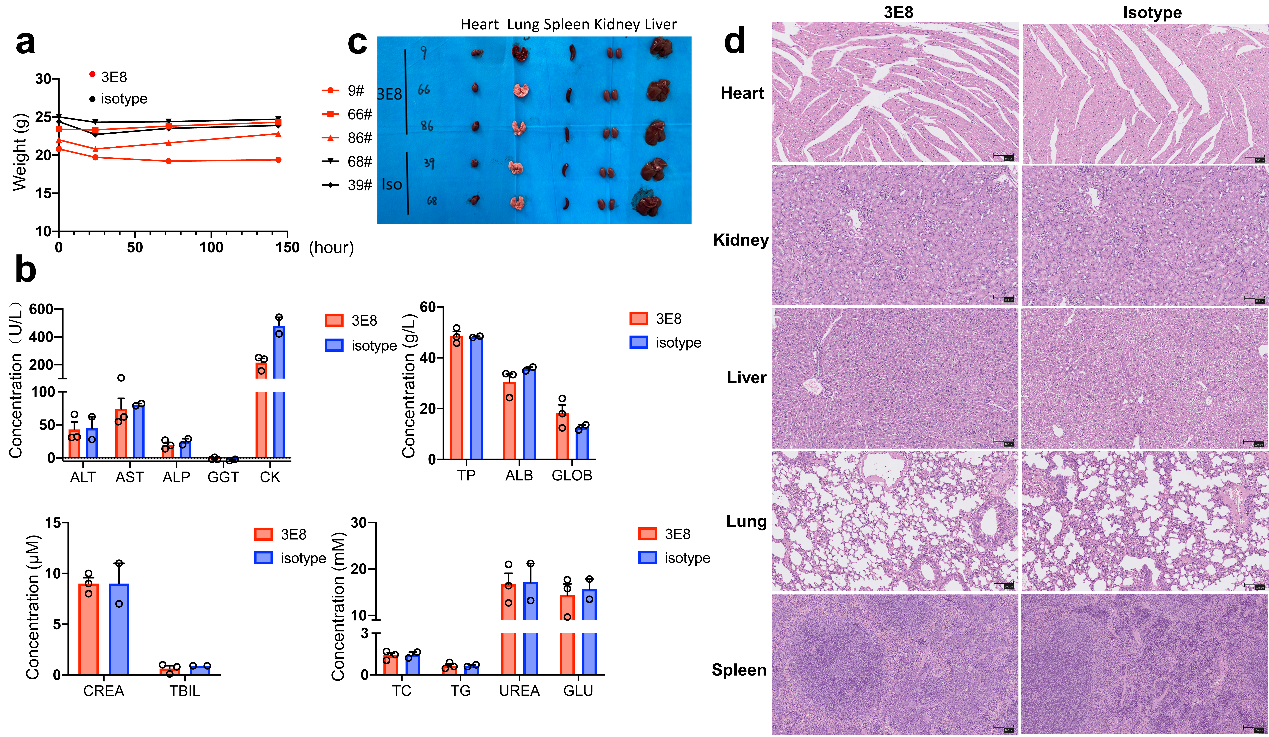


**Figure. S3.**

Toxicity studies of antibody 3E8 in human ACE2 “knock-in” mice.

**a** The body weights of the treated mice were measured at 0, 24, 72 and 144 h time points. **b** Blood biochemistry analysis showed the plasma concentrations of alanine transaminase (ALT), aspartate transaminase (AST), alkaline phosphatase (ALP), gamma-glutamyl transpeptidase (GGT), creatine kinase (CK), total protein (TP), albumin (ALB), globulin (GLOB), creatinine (CREA), total bilirubin (TBIL), total cholesterol (TC), triglycerides (TG), urea nitrogen (UREA) and glucose (GLU). The indices of GGT and TBIL in 3E8 group and isotype group were below the normal range, but lowered levels are not signs of toxicity. **c** Shapes and sizes of major organs including hearts, livers, kidneys, spleens and lungs from mice 7 days post treatment. The organs were dissected out after the mice were sacrificed by cervical dislocation and then washed with PBS to clean out the blood. Blood clogging occurred in the lung of mouse 9# during the dissection process, which was determined technical. The mice were otherwise normal. **d** H&E staining of hearts, livers, kidneys, spleens and lungs of treated mice. No obvious pathological changes were observed. The scale of ratio above represents 100 μm.


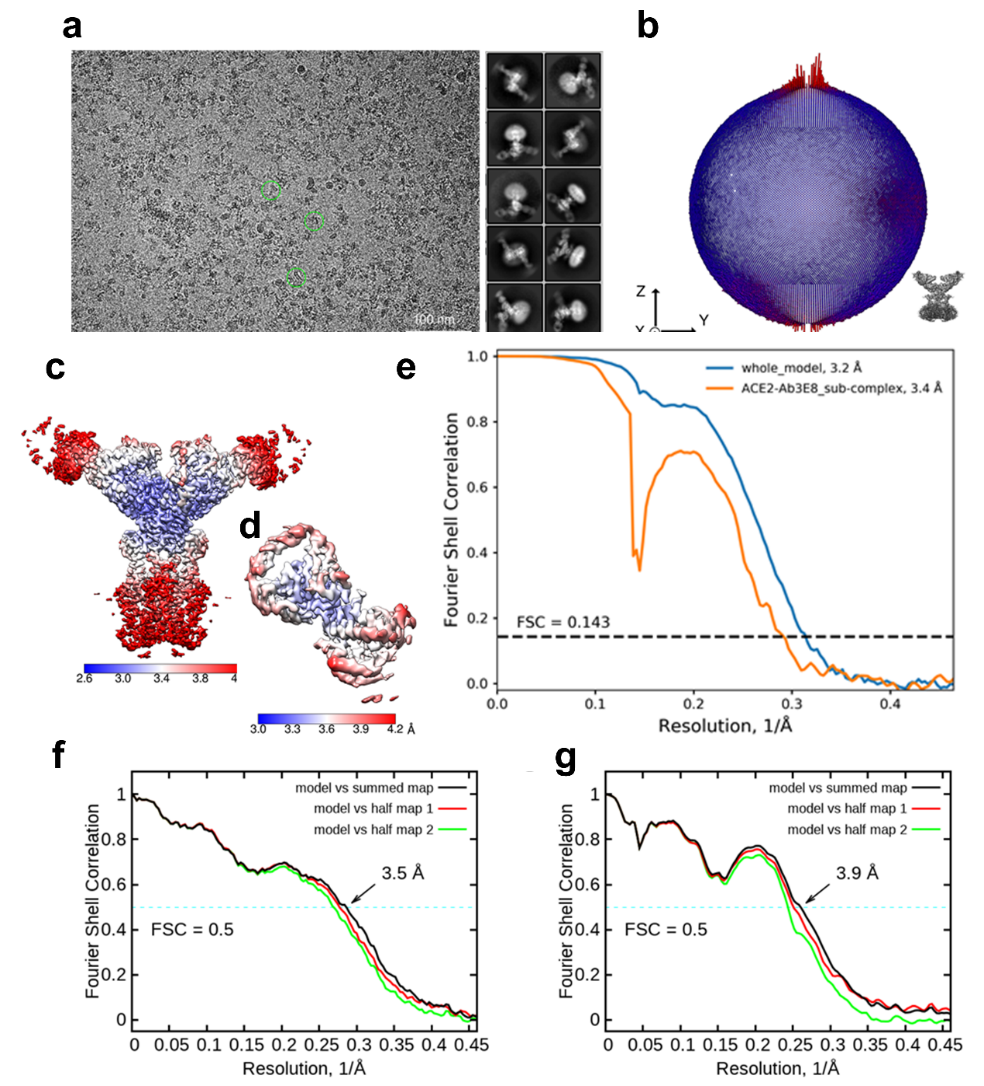


**Figure. S4.**

Cryo-EM analysis of 3E8 bound with ACE2-B^0^AT1 complex.

**a** Representative cryo-EM micrograph and 2D class averages of cryo-EM particle images. The scale bar in 2D class averages is 10 nm. **b** Euler angle distribution in the final 3D reconstruction of 3E8 bound with ACE2-B^0^AT1 complex. **c** and **d** Local resolution maps for the 3D reconstruction of overall structure and the 3E8-ACE2 sub-complex, respectively. **e** FSC curve of the overall structure (blue) and 3E8-ACE2 sub-complex (orange). **f** FSC curve of the refined model of 3E8 bound with ACE2-B^0^AT1 complex versus the overall structure that it is refined against (black); of the model refined against the first half map versus the same map (red); and of the model refined against the first half map versus the second half map (green). The small difference between the red and green curves indicates that the refinement of the atomic coordinates is not enough overfitting. **g** FSC curve of the refined model of the 3E8-ACE2 sub-complex, which is the same as the **f**.


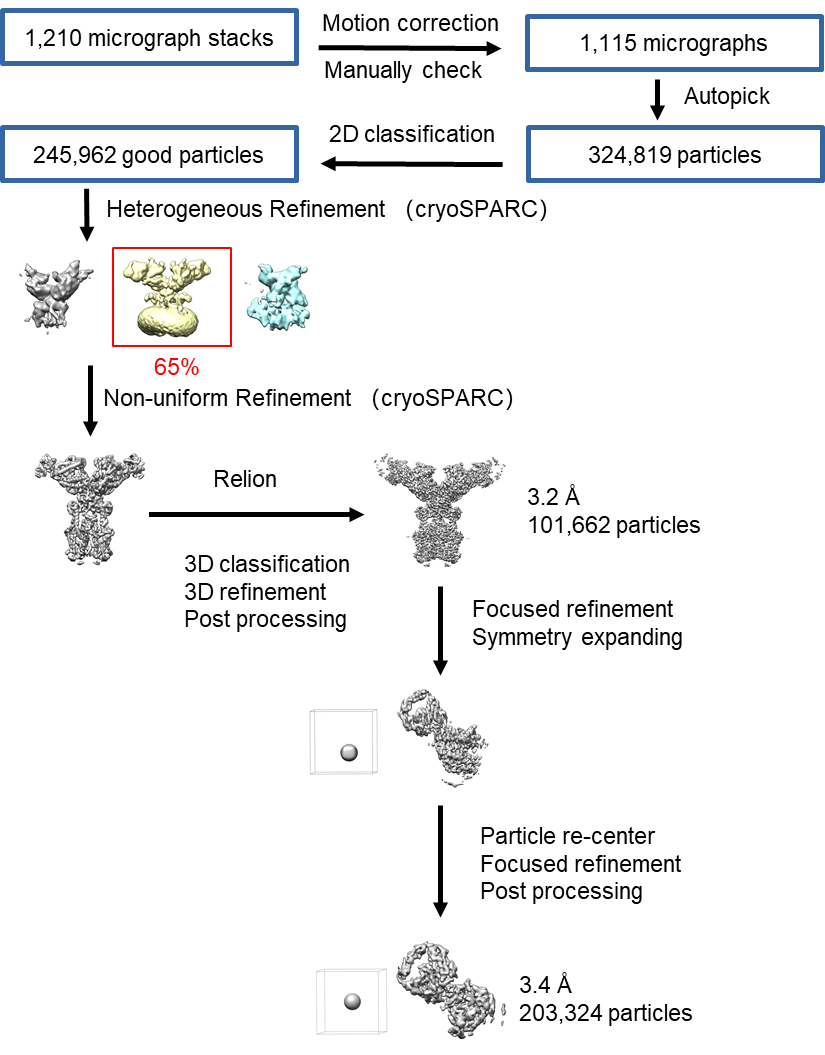


**Figure. S5.**

Flowchart for cryo-EM data processing. Please refer to the ‘Data Processing’ section in Methods for details.


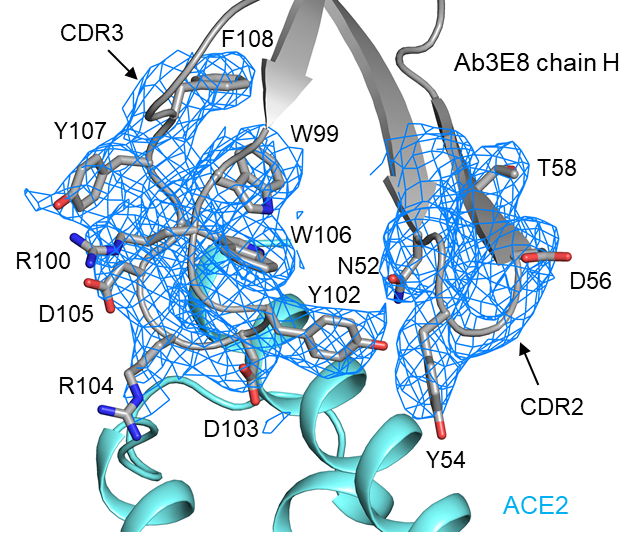


**Figure. S6.**

Representative cryo-EM map densities. Cryo-EM density of the interface between ACE2 and chain H of 4A8. The density is contoured at 10 σ.

**Table S1.** Cryo-EM data collection and refinement statistics.

| **Data collection** |  |  |
| --- | --- | --- |
| EM equipment | Titan Krios (Thermo Fisher Scientific) | |
| Voltage (kV) | 300 | |
| Detector | Gatan K3 Summit | |
| Energy filter | Gatan GIF Quantum, 20 eV slit | |
| Pixel size (Å) | 1.077 | |
| Electron dose (e-/Å2) | 50 | |
| Defocus range (μm) | -1.2 ~ -2.2 | |
| Number of collected micrographs | 1,210 | |
| Number of selected micrographs | 1,115 | |
| Sample | 3E8 bound with ACE2-B0AT1 complex | |
| **3D Reconstruction** |  |  |
|  | Whole model | Interface between 3E8 and ACE2 |
| Software | cryoSPARC/ Relion | Relion |
| Number of used particles | 101,662 | 203,324 |
| Resolution (Å) | 3.2 | 3.4 |
| Symmetry | C2 | C1 |
| Map sharpening B factor (Å^2^) | -90 | |
| **Refinement** |  |  |
| Software | Phenix | |
| Cell dimensions (Å) | 310.176 | |
| Model composition |  |  |
| Protein residues | 3,586 | |
| Side chains assigned | 3,586 | |
| Sugar | 38 | |
| Water | 4 | |
| Zn | 2 | |
| R.m.s deviations |  |  |
| Bonds length (Å) | 0.010 | |
| Bonds Angle (˚) | 1.027 | |
| Ramachandran plot statistics (%) |  |  |
| Preferred | 91.53 | |
| Allowed | 8.22 | |
| Outlier | 0.25 | |

**References and Notes**

1 Lei, J. & Frank, J. Automated acquisition of cryo-electron micrographs for single particle reconstruction on an FEI Tecnai electron microscope. *J Struct. Biol.* **150**, 69-80 (2005).

2 Zheng, S. Q. *et al.* MotionCor2: anisotropic correction of beam-induced motion for improved cryo-electron microscopy. *Nat. Methods* **14**, 331-332 (2017).

3 Grant, T. & Grigorieff, N. Measuring the optimal exposure for single particle cryo-EM using a 2.6 Å reconstruction of rotavirus VP6. *Elife* **4**, e06980 (2015).

4 Zhang, K. Gctf: Real-time CTF determination and correction. *J. Struct. Biol.* **193**, 1-12 (2016).

5 Zivanov, J. *et al.* New tools for automated high-resolution cryo-EM structure determination in RELION-3. *Elife* **7**, e42166 (2018).

6 Kimanius, D., Forsberg, B. O., Scheres, S. H. & Lindahl, E. Accelerated cryo-EM structure determination with parallelisation using GPUs in RELION-2. *Elife* **5**, e18722 (2016).

7 Scheres, S. H. RELION: implementation of a Bayesian approach to cryo-EM structure determination. *J. Struct. Biol.* **180**, 519-530 (2012).

8 Scheres, S. H. A Bayesian view on cryo-EM structure determination. *J. Mol. Biol.* **415**, 406-418 (2012).

9 Punjani, A., Rubinstein, J. L., Fleet, D. J. & Brubaker, M. A. cryoSPARC: algorithms for rapid unsupervised cryo-EM structure determination. *Nat. Methods* **14**, 290-296 (2017).

10 Rosenthal, P. B. & Henderson, R. Optimal determination of particle orientation, absolute hand, and contrast loss in single-particle electron cryomicroscopy. *J. Mol. Biol.* **333**, 721-745 (2003).

11 Chen, S. *et al.* High-resolution noise substitution to measure overfitting and validate resolution in 3D structure determination by single particle electron cryomicroscopy. *Ultramicroscopy* **135**, 24-35 (2013).

12 Trabuco, L. G., Villa, E., Mitra, K., Frank, J. & Schulten, K. Flexible fitting of atomic structures into electron microscopy maps using molecular dynamics. *Structure* **16**, 673-683 (2008).

13 Emsley, P., Lohkamp, B., Scott, W. G. & Cowtan, K. Features and development of Coot. *Acta Crystallogr D. Biol. Crystallogr.* **66**, 486-501 (2010).

14 Adams, P. D. *et al.* PHENIX: a comprehensive Python-based system for macromolecular structure solution. *Acta Crystallogr. D Biol. Crystallogr.* **66**, 213-221 (2010).
